# Supplementary material for: Are carbon emissions trading and green financial instruments synergistic? -Comprehensive quantitative research based on content analysis
Source: PLoS One. 2024 Mar 7;19(3):e0298601. doi: 10.1371/journal.pone.0298601 (PMC10919601; doi:10.1371/journal.pone.0298601)
Supplement: S1 Data — (ZIP) [file pone.0298601.s001.zip › Data/地级市Z_F工作报告.zip!/╔╜╢1⁄2/╟α╡║╩╨/└┤╫╘╣1⁄2╓┌║┼í╛┬φ┐╦╩2╛▌═°í┐_2011.docx]

各位代表：

　　 现在，我代表市人民政府向大会作工作报告，请连同《青岛市国民经济和社会发展第十二个五年规划纲要（草案）》一并审议，并请各位政协委员和其他列席人员提出意见。

　　一、“十一五”时期国民经济和社会发展回顾

　　“十一五”时期，是青岛发展史上极不寻常、极不平凡的五年。在中共青岛市委的坚强领导下，我们紧紧依靠全市人民，坚持以邓小平理论和“三个代表”重要思想为指导，深入贯彻落实科学发展观，坚决执行国家宏观调控政策，着力实施“环湾保护、拥湾发展”战略，保持了经济社会平稳较快发展，圆满完成“十一五”规划确定的目标任务。五年来，我们积极抢抓历史性机遇，努力克服各种严峻挑战，全面开创经济社会发展新局面，取得了来之不易的可喜成就。

　　——与2005年相比，全市生产总值连续迈上3000亿、4000亿和5000亿元的台阶，2010年达到5666亿元，年均增长13.8%；地方财政一般预算收入相继突破200亿、300亿和400亿元，2010年达到452亿元，年均增长21%；城市居民人均可支配收入由12920元提高到24998元，年均增长14.1%；农民人均纯收入由5806元提高到10550元，年均增长12.7%，分别高于“十一五”规划目标4.1个和2.7个百分点。服务业增加值占生产总值的比重达到46.4%，提高4.8个百分点。“十一五”节能减排目标全面完成。

　　——五年来，累计完成固定资产投资10321亿元，年均增长23%；规模以上工业总产值迈上万亿元台阶，2010年达到11450亿元，年均增长21.7%；高新技术产业产值突破5500亿元，占规模以上工业总产值比重达到47%。累计完成进出口总额2324亿美元，实际到账外资148亿美元，年均分别增长13%和13.5%。

　　——2010年，城镇化率达到66%，比2005年提高7.5个百分点；市区集中供热普及率达到62%，提高32个百分点；污水集中处理率达到85%，提高23个百分点。居民储蓄存款2963亿元，实现翻番。学前教育、义务教育和高中段教育入学率分别达到95%、100%和94%；政府办基层医疗卫生机构全部实施了基本药物制度，基本药物价格平均下降35%；城乡基层文化设施实现全覆盖。城镇登记失业率控制在4%以内。

　　回顾五年，我们主要做了以下工作：

　　（一）推动产业结构优化升级，现代产业体系初步形成

　　着力构建以服务经济为主的产业结构，加快推动重点产业从规模优势向技术优势转变。服务业呈现跨越发展新态势，增加值达到2630亿元，年均增长15.6%。引进金融机构64家，金融业增加值年均增长25.5%。港口吞吐量和集装箱吞吐量分别达到3.6亿吨、1200万标箱，空港旅客吞吐量突破1100万人次。旅游业总收入和接待国内外游客分别超过580亿元、4500万人次，崂山景区进入国家5A级旅游景区行列。大型商业设施面积达到380万平方米，是2005年的1.5倍。成为国家首批服务业综合改革试点城市。制造业呈现“7+5”发展新格局，规模以上工业企业突破6000家，制定了高端产业发展意见，培育了家电电子、石化化工等六个千亿级产业集群，船舶海洋工程等七大重点产业产值占工业比重超过85%。成为国家首批“两化融合”试验区和“三网融合”试点城市。现代农业呈现规模化发展新特色，“四个百万亩示范区”全面实施，粮食总产由315万吨提高到351万吨。

　　2008年下半年以来，为应对国际金融危机的严重冲击，根据国家和省的部署，迅速实施了扩内需、保增长的一揽子措施，安排总投资5000亿元的重点项目500个。城市地铁、董家口港区等一批项目开工建设。建立了市级机关领导干部联系企业制度，帮助解决企业发展和项目建设的突出问题。降低中小企业准入门槛，在融资担保、市场开拓、技术创新和员工培训等方面加大政策支持。帮助企业融资1730亿元、完成出口退税314亿元、减免各种税费100亿元，率先实现经济回升向好。

　　（二）强化自主创新节能减排，经济发展方式加快转变

　　实施“四个十”科技创新工程。与中科院全面加强战略合作，引进所属4家科研机构，青岛海洋国家实验室开工建设。强化企业技术创新主体地位，新增19个国家级企业重点实验室、工程技术研究中心等创新平台，国家级企业技术中心和创新型企业总数居计划单列市之首。新增中国驰名商标66件，取得高速动车、液晶模组等一批重大创新成果，主导或参与制订、修订国际标准9项。成为全国首个技术创新工程试点城市、国家创新型试点城市和知识产权示范城市。五年获得国家科技奖励48项，发明专利申请量、授权量均比“十五”时期翻了一番。实施“222引才工程”和“300海外高层次人才引进计划”，引进各类人才16万人。

　　推进“十大节能工程”和“碧海蓝天工程”。万元生产总值能耗下降22%，二氧化硫和化学需氧量排放量分别削减26.9%、18%。加强环境保护和生态修复，出台《青岛市海洋环境保护规定》，启动环湾流域治理工程，市区河道截污主干管网基本贯通，李村河、海泊河等整治效果明显，胶州湾生态湿地保护得到加强，近岸海域功能区水质达标率为87.5%，提高14.5个百分点。新建民用建筑全部达到节能标准。市区空气质量优良天数保持在330天以上。全市森林覆盖率和建成区绿化覆盖率分别提高5.2个、4.8个百分点，成为全国绿化模范城市。成功申办2014年世界园艺博览会，各项筹备工作全面展开。

　　（三）统筹区域协调发展，城乡面貌发生明显变化

　　不断提高科学规划、集约建设、精细管理水平。基础设施建设五年累计投资超过2000亿元，年均增长34%。海底隧道、海湾大桥主体贯通，青兰、青新高速等竣工通车，铁路青岛客运北站和青荣、海青铁路等项目进展顺利，市域“一小时经济圈”基本形成。制订出台蓝色经济区发展规划纲要，成功举办首届蓝色经济发展国际高峰论坛。新一轮城市总体规划修编完成。高新区实现扩区，创建国家创新型科技园区获得批准，胶州湾北部园区基础设施初具规模，通过ISO14001环境质量体系认证，45个过亿元项目开工建设。董家口港区开发全面启动，华能通用码头等项目实现简易投产。建成数字化城市管理新模式，市区铁路沿线区域整治和城市亮化初见成效。

　　新农村建设和县域工业化步伐加快。市级财政“三农”投入102亿元，比“十五”时期增加2.4倍。发放农业“四项补贴”17亿元，年均增长39%。主要农作物良种覆盖率达到99%，生产机械化水平达到80%。投资43.6亿元，完成农村供水、防洪排涝等五大水利工程。村庄“五化”完成90%。新建、改造农村住房14.5万套，修建农村残疾人住房5600套。规划建设了6个县域工业功能区，54户城区企业实施搬迁改造，17户竣工投产。县域工业产值占全市比重提高6.1个百分点。

　　（四）全面深化改革扩大开放，发展活力不断增强

　　重点领域改革进一步推进。国有经济行业分布由76个减少到48个。整合组建国信、华通、城建、交通、港口五个投资公司，规范了政府投融资管理。新一轮政府机构改革完成，文化广电新闻出版改革、文化单位转企改制、文化市场综合执法改革迈出实质步伐，农村综合配套改革试点和集体林权制度改革稳步推进，开发区综合配套改革全面实施。市中小企业公共服务中心建成运营。个体私营企业达到42万户，注册资本超过2300亿元，非公经济占生产总值的比重达到68.4%。

　　全方位开放格局进一步形成。强化定向招商，引进过亿美元项目20个、世界500强企业12家。各类经济园区利用外资占全市比重由42%提高到48%。机电产品和高新技术产品出口分别比“十五”时期增加1.9倍、2.4倍，新兴市场出口比重提高5.4个百分点。引进内资和企业境外投资分别增加1.7倍、4.1倍。青岛保税港区一期封关运营，西海岸出口加工区投入使用。口岸开放步伐加快，“大通关”成效显著。国际友好城市和友好合作关系城市达到56个。科技、教育、文化、卫生、体育、旅游等领域对外交流与合作迅速扩大，取得积极成果。对台工作全面开展，侨务工作取得新成效。

　　对口帮扶合作进一步加强。与西藏日喀则，贵州铜仁、安顺，湖北宜昌三峡库区和我省菏泽曹县等地开展了形式多样、成效显著的帮扶合作。四川汶川特大地震发生后，全力投入援川救灾各项工作，组织捐赠款物超过6亿元，帮助灾区人民恢复生产生活；先后派出3000多人的援建队伍，投资5.8亿元，建成23个援建项目，提前一年圆满完成对口支援北川灾后重建任务，充分展现了我们这座城市的爱心情怀！

　　（五）着力解决群众切身利益问题，城乡居民生活质量持续改善

　　保障和改善民生力度加大。五年来，市级财政用于社会民生投入达到366亿元，占同期地方财政一般预算支出的50%。投入138亿元，在城乡建设和改善群众生活方面安排实事50件。安排促进就业资金16.4亿元，新增就业创业181万人。设立大学生就业创业扶持资金，大学生就业创业率达到92.6%。五年来，两次提高城乡低保标准，三次提高最低工资标准，五次提高企业退休人员基本养老金。建立了物价上涨与城乡低保居民补贴联动机制。出台养老服务、残疾人社会保障和服务体系建设的实施意见，机构养老床位增加1.7倍，社区老年活动场所覆盖率达到96.8%。市政公用事业惠民力度不断加大。投资80亿元，建成500座换热站、3000公里供热管网，新增供热面积4160万平方米，占市区集中供热总量的60%。自来水一户一表、管道燃气气化率分别达到86%和75%。市级财力投入7.8亿元，新增更新公交车2774辆。

　　“两改”和保障性住房建设规模空前。2008—2010年保障性住房建设规划全面完成，财政直接投入、土地收益让渡和配套费减免超过100亿元，基本建立起多层次住房保障体系。建成6.2万套保障性住房，改造旧城区和城中村77个、实现回迁44个，整治旧住宅区16片，3.5万户低收入住房困难家庭实物配租配售率达到88%，市区10万户家庭住房条件得到改善。

　　（六）促进经济社会协调发展，社会保持和谐稳定

　　教育均衡发展步伐加快。五年来，财政性教育支出年均增长18.6%。率先实现城乡免费义务教育，统一了县域教师工资水平，理顺了市区义务教育管理体制。实施了中小学取暖、饮水设施更新和薄弱学校改造等工程。解决了12.3万外来务工人员子女入学问题。建成职业教育公共实训基地和22个市级校内实训基地。高等院校全日制在校生超过30万人。各类学校学生资助体系进一步健全。

　　医疗保障水平明显提高。投资42.7亿元，建成国医堂、妇女儿童医院、北部中心医院、西海岸医院等大型医疗设施，镇卫生院和村卫生室标准化率分别达到100%、79.5%。基本公共卫生服务惠及全民，医疗保障制度实现全覆盖。新农合为1200万人次报销医药费超过15亿元。疾病防控和应对突发公共卫生事件的能力不断增强。

　　文化强市战略加快实施。深入推进“人文奥运”工程，新一轮全国文明城市创建效果明显，涌现出一批全国文明单位、文明村镇和道德楷模。荣获5项全国“五个一”工程奖。志愿者队伍达到44.5万人。奥帆中心、青岛大剧院、体育中心等大型设施建成，图书馆、博物馆等44处公共文化场所免费开放。设立1亿元文化产业发展专项资金，文化创意产业增加值占生产总值比重达到7.7%。我市运动员在北京奥运会、广州亚运会和全国运动会上取得历史最好成绩。

　　人口、双拥等社会事业全面发展。人口与计划生育综合改革步伐加快，低生育水平保持稳定。第六次人口普查取得阶段性成果。“双拥”工作扎实开展，军地之间互办实事互解难题，优抚标准不断提高，国防动员、人民防空和民兵预备役建设成效显著，第六次荣获“全国双拥模范城”。妇女儿童、档案、仲裁和红十字、慈善事业稳步推进，民族宗教工作健康发展。

　　平安青岛建设不断加强。注重源头治理，健全社会治安动态防控体系，始终保持对违法犯罪的高压态势。加强了城乡和谐社区建设和管理服务。出台重大事项社会稳定风险评估办法，完善矛盾纠纷排查调处长效机制，形成“大调解”工作格局，一批多年积累的信访疑难问题得到解决。安全生产网格化监管全面推行，事故起数和死亡人数分别下降66.5%、46.7%。全国食品药品最安全城市创建工作稳步开展。公共安全应急管理水平不断提高，综合防灾减灾能力进一步增强。

　　（七）推进依法行政政务公开，服务型政府建设步伐加快

　　坚持办事讲程序、工作讲规则、决策讲民主，不断提高政府工作法制化、制度化、规范化水平。严格遵守国家法律法规，按照法定权限和程序行使职权。加强和规范政府行政决策、执行、监督程序，注重政务公开和阳光操作，恢复建立了城市管理规划委员会制度和专家咨询委员会制度，依法对城乡规划、大型基础设施建设、用地性质变更、项目容积率调整、财政专项资金安排、重大活动举办等实行合法性审查和集体讨论决定，切实维护其严肃性和权威性。修订了《青岛市人民政府工作规则》。自觉接受市人大及其常委会的法律监督和工作监督，主动接受市政协的民主监督，不断加强与各民主党派、工商联、无党派人士的对口联系。提请市人大常委会审议通过地方性法规24件，制定政府规章18件。组织开展第四轮行政审批事项清理，行政审批服务大厅和网上审批系统建成启用，审批效率平均提速70%。“五五”普法规划顺利完成。

　　及时回应社会热点问题，自觉接受公众监督。创新开展市政府部门“向市民报告、听市民意见、请市民评议”活动，建立健全政府网站、行风在线、政务服务热线、民生在线、纠风网、网络在线问政等互动平台，构建了公众评价政府的新机制。不断完善政府信息公开、新闻发布制度，电子政务和网上公共服务体系基本形成。

　　廉政建设得到加强。健全教育、制度、监督并重的惩治和预防腐败体系，建成23项重要行政权力网络运行和电子监察系统，公共资源交易大厅和综合电子监察平台投入使用。建立专项资金预算公开和绩效管理制度，财政专项资金使用管理更加规范。审计结果公告制度全面实施。扎实开展“小金库”治理、工程建设领域突出问题整治、中央扩大内需项目监督检查等专项工作。严肃查处了一批贪污贿赂、失职渎职等违法违纪案件。

　　各位代表！2010年，我们努力扩大应对国际金融危机的成果，经济社会保持了平稳较快发展。初步核算，全市生产总值比上年增长12.9%，地方财政一般预算收入增长20.1%，城市居民人均可支配收入和农民人均纯收入分别增长11.8%、14.1%，市十四届人大三次会议确定的目标任务全面完成。

　　一是围绕转变经济发展方式，着力优化投资结构、消费结构和出口结构。年初确定的总投资2828亿元的240个重点项目全部开工，华强文化科技产业基地等120个现代服务业项目进展顺利，南车高速列车产业化基地等79个项目竣工投产，环湾大道全线通车。总投资1138亿元的361个工业振兴项目，赛轮1000万条子午胎等120个项目竣工投产。城乡商业零售网点达到33万处，社会消费品零售总额超过1900亿元，增长18.7%。对外贸易和利用外资全面恢复到金融危机前水平，进出口总额和实际到账外资分别增长32%、52.8%。成功举办青岛国际帆船周、东亚经济交流推进机构第四次大会等重要活动。

　　二是围绕提高城市创新能力，着力推进科技、教育和人力资源协调发展。全面实施《高新技术高端产业创新发展工程建设规划》，组建14家产业技术创新战略联盟，青岛工业技术研究院挂牌运营，国家大学科技园通过认定，青岛国家质检中心基地一期建成启用。市、区（市）两级机关全面实现软件正版化。提高并统一城乡义务教育公办学校生均公用经费标准，率先建立高中段公办学校生均公用经费财政预算内拨款制度，中小学生均经费居计划单列市首位。投资1.2亿元，建成2.5万平方米的引进高层次人才公寓。

　　三是围绕保障和改善民生，着力提高城乡居民收入、医疗和住房保障水平。市级财政用于社会民生投入超过110亿元，比上年增长26%。年初确定为城乡居民重点办好的9件实事全部完成。去年1月，提高企业退休人员基本养老金，月人均达到1561元，增长12.6%；5月，提高最低工资标准，七区和五市分别达到920元、760元，增长21%、22%；12月，新增财政资金4.76亿元，将全市企业退休人员的取暖补贴由人均80元提高到1100元。下半年，针对居民生活必需品价格较快上涨，出台了保证市场供应、保持物价稳定、保障群众生活的20条措施，四次启动物价上涨与城乡低保居民补贴联动机制。医药卫生体制改革全面展开，城乡居民基本医疗保险补助额提高到每人每年120元，开展了创建“人民满意的医疗卫生机构”活动。保障性住房建设步伐加快，竣工和配租配售住房1.7万套，20个“两改”项目开工建设，河马石2285套公共租赁住房小区建成使用。文化青岛建设成效显著，全国文化体制改革会议在我市隆重举行。

　　四是围绕促进社会稳定，着力抓好社会治安、公共安全、生产安全。积极化解各种社会矛盾，严厉打击了一批黑恶势力和违法犯罪活动。组建了校园专职保安队伍。开展“安全生产基层基础年”活动，安全生产形势保持稳定。投入5000万元，提高了消防设施和装备水平，突出了高层建筑和建筑工地等重点领域的消防监管，加强了重大灾害事故应急救援综合演练。

　　各位代表！过去的五年，在跌宕起伏的发展环境中，我们经受住稳定政局凝聚人心、抗击浒苔自然灾害、应对国际金融危机等一系列重大考验，成功承办北京奥运会、残奥会帆船比赛等一系列重大活动。五年来，我们始终坚持解放思想，以转变观念推动创新发展，以调整思路促进结构优化，以改革开放增创竞争优势；始终坚持科学发展，注重产业转型升级，注重城乡统筹协调，注重资源节约环境友好；始终坚持以人为本，真心实意关心群众疾苦，尽心竭力办好民生实事，让改革发展成果惠及城乡所有居民；始终坚持依法行政，自觉接受社会监督，不断提高公信力、执行力，努力建设人民满意的服务型政府；始终坚持奥运带动，完善基础设施，创新体制机制，打造“帆船之都”新品牌，全面提升城市国际化水平和影响力，为“十二五”发展奠定了坚实基础！

　　五年的巨大变化，积累的宝贵经验，是在党中央国务院和省委省政府科学决策、坚强领导下不断开创的，是在各行各业和社会各界坚定信心、团结拼搏的实践中共同造就的，是在历届市领导班子工作的基础上与时俱进、开拓创新持续形成的。在这里，我代表市人民政府，向辛勤工作在各条战线的广大市民和外地来青建设者，表示衷心的感谢！向各位市人大代表、政协委员和各民主党派、工商联、无党派人士，向离退休老同志、各人民团体，向人民解放军驻青部队、武警官兵、公安干警和中央、省驻青单位，向所有关心支持青岛发展的海内外朋友们，致以崇高的敬意!

　　回顾过去的五年，我们也深刻地认识到，政府工作存在诸多不足，经济社会发展仍面临不少困难和问题。主要是：一些政府工作人员法治意识、责任意识、服务意识不强，行政不作为、乱作为等现象依然存在，工作作风和综合素质有待提高；政府部门的战略视野、工作标准与建设现代化国际城市的要求仍有较大差距，社会管理、公共服务的体制机制和方式方法亟待改革创新。可持续发展的资源环境约束不断强化，产业结构调整任务艰巨，自主创新能力有待提高，战略性新兴产业亟待培育；县域经济发展不快，非公经济比重不高，国有经济布局过宽，一些领域的改革进展缓慢；支柱性产业大项目偏少，学科带头人和拔尖人才缺乏，集聚国内外优质资源的能力较为薄弱；城乡居民总体收入水平不高，持续增收渠道不宽，关系群众切身利益的住房、教育、医疗、社会保障、养老服务等方面存在不少亟待解决的问题；文化软实力与经济社会发展不够协调，城市文明程度需要进一步提升，社会正气需要进一步弘扬；安全生产形势不容乐观，维护和谐稳定压力增大。对于这些问题，我们一定以高度负责、攻坚克难的精神，采取有效措施努力克服和认真解决。

　　二、“十二五”时期的总体要求和目标任务

　　各位代表！“十二五”时期，世情、国情、市情都会发生新的变化。做好今后五年的工作，必须准确把握经济社会发展的阶段性特征，深刻认识面临的历史机遇和严峻挑战，切实增强前瞻性、科学性和创造性，牢牢把握发展的主动权。

　　我们清醒地看到，当前，世界经济正在经历深度变革调整，经济全球化深入发展，科技创新和产业升级孕育重大突破；我国工业化、信息化、城镇化、市场化、国际化加速推进，市场潜力巨大、发展空间广阔；山东半岛蓝色经济区上升为国家战略，区域经济发展将形成新的格局。我们清醒地看到，加快转变经济发展方式更加紧迫，长期形成的结构性矛盾和粗放型增长方式，已经到了只有加快转型才能实现长期可持续发展的关键时期。我们清醒地看到，改革发展稳定的任务更加艰巨，改革的重点已经从经济领域向其他领域拓展，触及的矛盾更深，涉及的利益更复杂，推进的难度更大，加快创新体制机制已进入攻坚阶段。我们清醒地看到，社会结构深刻变动，利益格局深刻调整，思想观念深刻变化，群体诉求日趋多样，对加强社会建设、创新社会管理服务提出了更高要求。我们必须紧紧抓住战略机遇期，积极作为，科学务实，主动适应环境变化，有效化解各种矛盾，加快转方式调结构，促进经济平稳较快发展，不断提高民生保障能力，保持社会和谐稳定。

　　“十二五”时期，经济社会发展的总体要求是：高举中国特色社会主义伟大旗帜，以邓小平理论和“三个代表”重要思想为指导，深入贯彻落实科学发展观，以科学发展为主题，以加快转变经济发展方式为主线，按照世界眼光、国际标准，发挥本土优势，深入实施“环湾保护、拥湾发展”战略，坚定不移地推进改革开放，全面提高自主创新能力，着力保障和改善民生，切实加强和创新社会管理，加快向富强文明和谐的现代化国际城市迈进，为经济文化强省建设作出更大贡献。

　　“十二五”时期，经济社会发展的主要预期目标是：在优化结构、提高效益、降低消耗、保护环境的基础上，全市生产总值年均增长11%，地方财政一般预算收入年均增长15%左右，服务业增加值占生产总值比重达到57%；全社会研发投入占生产总值比重达到2.5%；城镇化率提高到75%左右；单位生产总值能耗持续降低，主要污染物排放总量明显减少，森林覆盖率、建成区绿地率均达到40%；公共服务体系和社会保障体系更加完善，努力实现城乡居民收入增长和经济发展同步、劳动报酬增长和劳动生产率提高同步，城镇登记失业率控制在4%以内。

　　根据上述总体要求和预期目标，突出把握好六个重点：

　　（一）加快推进经济结构战略性调整，努力实现高端引领、内需驱动的率先发展。坚持高端化、集约化发展，加快构建现代产业体系。以扩大内需为重点，不断优化投资结构，促进经济增长向依靠消费、投资、出口协调拉动转变。突出发展高端服务业，着力培育特色聚集区、骨干企业和名牌产品。以新一代信息技术、节能环保、新能源、新材料等为重点，尽快把战略性新兴产业培育成支柱性产业。深入实施质量兴市、品牌和标准化战略，积极运用新兴网络和信息技术，推进“两化融合”和“三网融合”，企业和社会信息化应用水平全面提高。大力发展高产、优质、高效、生态、安全农业，促进农业生产经营专业化、标准化、规模化、集约化，县域经济实力明显增强。

　　（二）加快推进城乡区域一体化，努力实现七区协调、城乡互动的统筹发展。坚持把统筹城乡区域发展和新农村建设作为全面建设小康社会的根本要求，实施新型城镇化战略，加快建设五市城区和重点中心镇，实现七区公共服务一体化，构筑现代化城市发展新格局。实施重点区域带动战略，全力建设蓝色经济区，优化调整陆海空间布局，重点突破一批海洋核心技术，切实保护海洋生态环境，建成国际一流、国内领先的现代海洋产业聚集区。加快海港、空港、信息港、综合路网及市政设施建设，提升城市综合服务功能。高新区生态科技城、董家口现代化港城初具规模，世园会主题园区及周边区域成为绿色休闲城市的标志区。

　　（三）加快推进以保障和改善民生为重点的社会建设，努力实现富民惠民、公平正义的和谐发展。坚持不断完善保障和改善民生的制度安排，推动基本公共服务均等化，使困难群众生活更有保障，老年群体生活得到更多关怀，全体居民生活普遍改善。实施更加积极的就业政策，合理调整收入分配关系，优化扩大就业创业、增加劳动收入的政策条件和环境，建成国家级创业型城市。健全覆盖城乡的社会保障、基本医疗、养老救助等服务体系，推进社会保障制度整合衔接，在扩大覆盖范围、提高保障水平和制度统一方面实现突破。加大保障性住房建设力度，多渠道筹措公共租赁住房，全面完成城中村改造。强化政府社会管理职能，更新社会管理理念，创新社会管理体制，整合社会管理资源，形成科学有效的利益协调、诉求表达、矛盾调处和权益保障机制，提高社会管理的科学化水平。落实计划生育基本国策，促进人口规模、结构、素质协调发展。始终把保护人民生命财产安全放在首位，构建公共安全和防灾减灾体系，营造社会安定、百姓安宁的良好局面。

　　（四）加快推进城市软实力建设，努力实现科教先进、文明进步的创新发展。坚持科技是第一生产力、人才是第一资源，全面落实中长期科技、教育、人才发展规划纲要。加快城市创新体系建设，实施重大科研项目攻关，深化产学研结合，加强知识产权保护。办好人民满意的教育，统筹城乡教育资源均衡配置，大力发展各级各类教育，基本实现教育现代化。实施人才优先发展战略，构筑更加科学合理、自由宽松的人才制度环境，建设区域性人才高地。围绕文化青岛建设，提高全社会文明素质，建成覆盖城乡的公共文化服务体系。发展哲学和社会科学，做好科普工作。推动文化创新，发展新的文化业态，把文化产业培育成重要的支柱产业。

　　（五）加快推进生态文明建设，努力实现资源节约、环境友好的绿色发展。坚持环保优先、可持续发展理念，认真贯彻节约资源和保护环境基本国策，强化节能减排，完善有利于绿色发展的政策措施。加快发展循环经济、低碳技术，壮大节能环保产业，形成资源节约、环境友好的生产方式和消费模式。加强城市管理，形成安全、整洁、有序、法治的管理格局。大力开展植树造林，改善城乡生态环境，建成国家生态园林城市。

　　（六）加快推进改革开放步伐，努力实现充满活力、互利共赢的开放发展。坚持不懈地创新体制机制，深化重点领域、关键环节改革，建设法治政府和服务型政府。加强国有企业和国有资产经营公司改革，优化国有经济布局。深入实施中小企业成长工程，营造各类市场主体公平竞争的发展环境。扩大全方位开放，促进区域合作互利共赢，积极拓展国内外交流合作的广度和深度，全面提高城市国际化水平。

　　各位代表！今后五年，我们将紧紧围绕科学发展的主题主线，善于在变化的形势中把握机遇，勇于在激烈的竞争中增创优势，努力完成本次会议和“十二五”规划纲要确定的各项目标任务，率先全面建成小康社会，率先建成创新型城市，使经济社会发展更加协调、更具活力，城乡居民生活更加幸福、更有尊严！

　　三、2011年的主要工作

　　今年，是实施“十二五”规划的第一年。我们将全面落实国家宏观调控政策，积极应对复杂多变的经济形势，努力实现良好开局，以优异成绩迎接中国共产党成立90周年。

　　经济社会发展的主要预期目标是：生产总值增长12%左右，地方财政一般预算收入增长15%，城乡居民收入均增长12%左右，城镇登记失业率控制在4%以内，居民消费价格涨幅控制在4%左右。人口自然增长率控制在3.9‰以内。全面完成国家和省下达的节能减排各项目标。

　　重点做好十个方面的工作：

　　（一）保持经济平稳健康运行

　　促进消费快速增长。继续落实好便民惠民各项消费政策，着力扩大商品消费。创新传统流通模式，发展新型消费业态，规范和扶持品牌连锁店等新型零售、餐饮业发展，推动商业、旅游、文化等多业联动，打造“青岛之夏”购物品牌。加强商业中心建设，提升商贸服务业集聚区功能，完善以物流配送、电子商务为特色的市场流通体系。力争社会消费品零售总额增长16.5%。

　　保持投资稳定增长。安排总投资3588亿元的重点项目260个，其中社会民生、服务业、技术创新项目占80%以上，年内确保80个项目竣工投产。完善鼓励和引导民间投资健康发展的配套政策，最大限度放宽民间投资准入领域和条件。争取固定资产投资增长18%，民间投资比重达到70%左右。

　　努力遏制物价过快上涨。严格执行国家和省出台的政策措施，落实“菜篮子”市长负责制，抓好“菜园子”工程建设。做好粮、油、肉、菜等大宗商品储备，提高市场应急保障能力。强化价格异常波动监测预警，加强市场监管，严厉打击价格违法行为。将物价上涨与城乡低保居民补贴联动机制扩大到五市。

　　切实加强房地产市场调控。全面落实国家房地产调控政策，加大中低价位、中小套型住房供给，严格实行住房限购政策，严格落实差别化住房信贷措施，严格管理住房用地供应，严格规范房屋交易秩序，遏制投资投机性购房，促进房地产市场平稳健康发展。

　　（二）加大产业结构调整力度

　　突出发展现代服务业。出台服务业综合改革试点实施方案和扶持政策。有重点地引进金融机构，培育地方法人金融机构，发展村镇银行、小额贷款公司，优化金融生态环境。加大力度推动企业上市，提高经营性国有资产证券化率，争取高新区纳入新三板扩大试点范围。做大做强软件产业、服务外包产业。港中旅海泉湾度假城等重点旅游项目建成开业。创建全国旅游标准化示范城市，完善旅游公共服务体系，努力开拓国内外旅游市场。加快发展总部经济和会展经济，办好中国国际消费电子博览会、国际帆船周、青岛国际啤酒节等品牌节会。

　　大力发展先进制造业和战略性新兴产业。推进信息化和工业化深度融合，推动产业振兴、技术改造，实施300个产业振兴项目和110个战略性新兴产业项目。制定战略性新兴产业激励政策，围绕新能源、新材料、生物医药等重点领域，集中引进一批大型骨干项目，实施一批重大应用示范工程，规划建设一批产业聚集区。

　　（三）推进农业现代化、县域工业化和新型城镇化

　　创建现代农业示范区。进一步加大对粮食生产的扶持力度，全面落实各项补贴奖励政策，抓好抗旱保苗，确保人畜饮水安全，力争粮食稳定增产。实施蔬菜、畜牧等五大产业振兴规划，加快花生、果茶两个百万亩示范区和平度国家现代农业示范区建设，培育一批设施农业、规模农业、特色农业和农副产品加工出口基地。加强农业科技创新和技术推广，发展农民专业合作社、家庭农场，提高农业产业化经营水平，培育一批具有较强竞争力的龙头企业。从今年起，把水利摆在基础设施建设的优先领域，把农田水利作为农村基础设施建设的重点任务，加大政府投入，加强骨干水利工程、河道治理和农田水利基本建设，完善城乡供水保障体系，形成城乡“大水网”。

　　加快县域工业化进程。优化县域产业布局，引导符合条件的企业和项目向产业园区集中，新启动15户老城区企业向县域工业功能区搬迁集聚，配套引进发展上下游关联企业。着力提高平度、莱西工业化水平，培育规模以上工业企业，促进县域中小企业发展。

　　实施新型城镇化战略。围绕新农村建设，完成市域城镇体系规划，提高五市驻地规划建设水平，优先安排与农民生产生活相关的基础设施和环境建设，加强郊区路域综合治理，做好道路、供排水管网、污水处理、垃圾收集等基础设施配套。推进国家级和市级小城镇发展改革试点。在城镇化建设中，切实保护农民的土地权益，尊重农民在进城和留乡问题上的自主选择权。

　　做好农村综合改革。加快农村社区股份合作和土地股份合作，深化集体林权制度配套改革，做好产权确权发证、森林资源评估等工作。扩大农业政策性保险试点范围。开展农村土地综合整治，完善集体经营性用地流转和宅基地管理机制。推进“乡财县管”改革试点。

　　（四）加快重点区域规划建设

　　高起点做好城乡规划。完成高新技术产业新城区、董家口港城的总体规划，开展温泉新城总体规划编制，深化环胶州湾东岸老工业区、四方欢乐滨海城、李沧交通商务区、红岛片区等区域的修建性详规及生态规划。完善市域交通、市政基础设施建设规划，完成轨道交通规划线网修编，做好地铁沿线、重庆路、世园会周边等区域的规划控制。合理调整盐田规划，科学有序开发利用盐田资源。

　　高水平建设蓝色经济区。认真落实《山东半岛蓝色经济区发展规划》，实施蓝色经济发展试点方案。开工建设国家深海基地，加快海洋国家实验室、海洋科考船等创新载体建设，建立蓝色经济重点项目库，加强海洋资源与环境保护技术开发，规划一批海洋自然保护区、湿地保护区和海岛保护区。办好2011年蓝色经济国际高峰论坛。我们一定要充分发挥青岛海洋科研教育优势，加强与半岛城市密切合作，在海洋科研创新、优势产业培育、陆海生态保护、国际交流合作等方面实现突破，率先建立现代海洋产业体系。

　　高质量推进重大基础设施建设。确保海底隧道、海湾大桥及接线工程主线通车，快速路三期、双高路等项目完工。加快建设青荣、青连、海青铁路和地铁一期、铁路客运北站、新疆路快速路等工程。开工建设青龙高速、重庆路快速路、福州路打通、世园大道等项目。做好新机场项目规划选址工作。

　　高标准打造重点功能区。积极推动七区市政设施、公共服务一体化发展，着力提升北部城区功能。统筹高新区“一区多园”建设，继续加大基础设施投资力度，规划建设国家大学科技园主园区、蓝色生物医药产业园、中德生态园，实施“3+1”主导产业行动计划，尽快形成产业竞争优势。加快董家口港区开发，完成西防波堤、进港航道等基础工程，做好国家一类口岸开放工作。推进青岛保税港区二期封关运营和功能区建设，深化经济技术开发区综合配套改革试点。支持黄岛生态智慧城、城阳航空城、崂山科技城、胶州服务外包科技城、市北啤酒文化休闲商务区等建设。

　　各位代表！2014年世界园艺博览会将在青岛举行，这是继2008年奥帆赛、残奥帆赛之后我市承办的又一国际盛会。我们将以“世界一流、中国时尚、山东特色、青岛品牌”为目标，坚持高起点、高质量、高标准，科学规划设计主题园区，加快基础设施及配套建设，带动提升北部城区功能和人居环境，努力举办一届世界水平、国际标准的园艺盛会！

　　（五）深入实施科教兴市和人才强市战略

　　加快建设创新型城市。做好国家技术创新工程试点工作。着力引进大院大所，培育国家级创新平台，推动建设市工业技术研究院。鼓励高校、科研院所与企业产学研合作，构建产业技术创新战略联盟，建设高新技术项目产业化基地。抓好1000项技术创新项目，开展物联网、节能减排、海洋防腐等技术应用示范。制定《青岛市科技进步条例》。支持发展风险投资、创业投资基金，积极培育科技中介机构。

　　坚持教育优先发展。完善教育经费保障机制，提高全市预算内教育经费占财政支出比重。抓好中小学校标准化建设，完成100万平方米校舍抗震加固工程，开工建设10所新建小区配套学校。将学前教育经费列入财政预算，提高公办幼儿园比重，加大对普惠性民办幼儿园的扶持。免除特殊教育学生在校学习生活费用。做好国家高等职业教育综合改革试点，筹建青岛远程教育中心。加强与高校的合作共建，积极稳妥地推进山东大学青岛校区的规划建设。各级各类学校要更加注重素质教育，保障每个学生健康成长。全社会要更加关心支持教育发展，营造尊师重教的浓厚氛围。

　　努力打造人才高地。深化“300海外高层次人才引进计划”和“急需高层次人才引进计划”，实施“443引才工程”，在现代产业、蓝色经济、城市规划建设管理和教育、文化、卫生等领域，引进一批领军人才和团队。重视创新型科技人才和高技能人才培养，特别要扶持创业人才和培养使用青年人才，最大限度地激发创新创造活力。完善居住证制度，促进人才柔性流动。

　　（六）全面加强生态文明建设

　　积极推进节能减排。完善合同能源管理，广泛开展建筑工程、交通运输、公共机构和居民生活等领域节能示范创建活动。建设李村河上游污水处理厂，加大胶州湾流域污染点源治理力度。做好机动车尾气、扬尘污染综合防治工作。强化节能减排目标责任制，健全污染减排统计、监测、考核体系。

　　大力发展循环经济。在17个重点行业开展清洁生产对标达标活动，对100家重点企业实施强制性清洁生产审核。创建石化产业国家级循环经济示范区。鼓励支持废旧汽车、家电、容器包装回收利用等静脉产业发展，扩大建筑废弃物资源化综合利用、新能源技术开发应用。重视开展低碳项目国际合作。倡导和鼓励绿色消费。

　　切实加强环境保护。推进“十绿”工程，加快太平山中央公园、浮山果艺生态园规划建设。提高数字化城市管理水平，开展“视觉污染”整治集中行动，做好汽车尾气、铁路两侧、山头景观等综合整治，继续实施过城河道、违法建筑、门头广告等专项治理，做好环湾大道、海湾大桥接线两侧环境绿化。深化环卫管理体制和运行方式改革。保护好胶州湾、大沽河和莱西姜山等湿地生态系统。探索建立环境污染责任保险制度。

　　（七）着力保障民生和发展社会事业

　　各位代表！新的一年，我们将顺应全市人民过上更好生活新期待，不断完善基本公共服务体系，把改善民生的各项工作做得更好，使改革发展成果更加均衡地惠及城乡居民！

　　重点做好七个方面的工作：一是努力增加居民收入。引导企业完善职工工资收入正常增长机制，探索建立企业效益与职工工资增长比例挂钩制度，继续提高企业最低工资标准。实施农民创业致富工程，建立促进农民持续增收的长效机制。二是千方百计扩大就业。突出以创业带动就业，新增城乡就业30万人，扶持自主创业1万人。三是提高社会保障水平。将企业退休人员基本养老金水平提高13.2%，月平均达到1767元。在全省率先实现城乡居民养老保险制度全覆盖，为88万60岁以上居民发放基础养老金。实施医疗、失业、生育、工伤四项保险市级统筹，推进城镇基本医疗保险门诊统筹试点。完善城乡一体的医疗救助制度，适时提高城乡低保标准，落实优抚对象、孤残儿童等群体的补贴政策。四是加大保障性安居工程建设力度。启动2011—2013年住房保障发展规划。全年开工1万套保障性住房、1万套限价商品房。开工“两改”项目46个，惠及居民2.5万户。新建、改造农村住房6万套。五是加快医疗卫生改革发展。将新农合和城镇居民医保补助标准提高到每人每年200元，政策范围内的住院费用支付比例达到70%左右。健全基本药物制度，抓好即墨市公立医院改革试点和市第三人民医院等重点医疗项目。加快基层卫生服务体系建设，进一步提高医疗服务质量，完善医患沟通和纠纷调解机制。六是提高养老救助服务能力。新增社区养老互助点400个、机构养老床位2600张。实施社会福利院、优抚医院、儿童福利院整体改造，新增残疾人托养床位1000张。建设市老年活动中心。七是改善居民生活环境。市区实现天然气双气源供应，新增供热面积900万平方米。改造居民二次供水设施178处，新建改建垃圾收集站40座，更新垃圾收集车400辆。新增、更新环保公交车1200辆。加强市区主要道路设施配套和调控，做好重点区域公用停车场规划建设，缓解日趋紧张的堵车和停车压力。确保完成向城乡居民承诺的12件实事。

　　大力发展各项社会事业。深化社区人口和计划生育综合管理，推进技术服务机构建设。继续做好第六次人口普查工作。增强全民国防意识，加强民兵预备役建设，全力为部队办实事解难题，争取第七次荣获“全国双拥模范城”。认真贯彻党的民族宗教政策，促进民族团结进步，依法管理宗教事务。深入实施妇女儿童发展纲要，重视发展慈善和社会公益事业。做好地方志编纂，推动档案、气象、地震、人防、仲裁、红十字等事业协调发展。

　　加强和创新社会管理。坚持人民的主体地位，发挥群众参与的基础作用，在思想上尊重群众，感情上贴近群众，工作上依靠群众，努力开创新形势下社会管理的新局面。建立完善适应不同群体、多元化的新型社会管理和服务机制，统筹协调各方面利益关系，保障人民群众安居乐业。重视各类社会组织和队伍建设，促进基层管理服务力量整合联动，切实维护群众合法权益，增强居民对社区的认同感和归属感。加强和完善信息网络管理，健全网上舆论引导机制。完善重大事项社会稳定风险评估机制和社会矛盾多元调解机制，畅通群众利益诉求渠道，形成源头防范、受理疏导、帮助解决的信访工作新格局。支持行业协会等民间组织依法参与社会管理。加强基层法律服务、法律援助和司法救助。

　　深入推进平安青岛建设。强化社会治安综合治理，严厉打击刑事犯罪。重视应急知识普及，做好应急预案演练，建立防灾减灾预警系统，加强海上溢油应急联动和物资储备，提升应对突发事件和自然灾害的快速反应能力。完善安全生产网格化监管机制，落实各级政府、行业监管责任和企业主体责任，突出道路交通、消防安全、建筑施工、危险化学品、市政管网等领域的安全监管。建设国家质检中心基地二期工程。加强食品药品安全体系建设，完善监督制度和管理机制。开展肉类蔬菜流通追溯体系试点工作，年底前市区100%的蔬菜批发市场、70%的零售市场建成质量安全追溯体系。

　　（八）加快文化青岛建设

　　提高市民文明素质和城市文明程度。以社会主义核心价值体系构筑精神家园，实施“四心、四德”工程，大力弘扬社会正气，增强社会诚信，广泛开展志愿服务。深入推进“文明交通行动计划”。做好未成年人社会课堂和乡村少年宫等场所的建设管理。深入开展“扫黄打非”斗争，加强文化市场综合执法，不断净化社会文化环境。全面开展“文明城市创建年”活动，争取以优异成绩荣获全国文明城市称号。

　　繁荣发展文化事业和文化产业。以筹备第十届中国艺术节为契机，实施文化创作精品工程和文化惠民工程，大力发展新闻出版、广播电视、文学艺术事业，鼓励创作更多思想深刻、艺术精湛、具有青岛特色的优秀文艺作品。启动市科技馆、文化中心等重点设施建设。办好第三届国际小提琴比赛、青岛音乐节、全民读书月等活动。做大做强文化产业，推进中国电影交易中心、国际影视演艺中心等重点项目建设，加快发展新媒体、动漫等新兴文化产业，推动传统文化产业转型升级。按照创新体制、转换机制、面向市场、增强活力的原则，深化文化体制改革。加强历史文化名城保护和利用，完成第三次全国文物普查。

　　提高全民健身和竞技体育水平。完善城乡居民健身设施网络，提高足球、帆船等运动项目在青少年中的普及率。加快足球训练中心等工程建设，健全体育中心设施功能。办好第12届苏迪曼杯国际羽毛球锦标赛，争取在第七届全国城市运动会上取得优异成绩。

　　（九）深化改革攻坚，提升开放型经济发展水平

　　营造富有效率、更具活力的体制机制。国有经济分布缩减到45个行业。实行国有企业财务风险动态监控和预警分析，实施全面预算管理。调整完善国有投资公司管理体制和运行机制，规范投融资行为，增强发展能力。抓好中小企业三级服务平台建设，加强信息咨询、信贷融资、法律援助、技能技术培训等服务，加大对小型微型企业的扶持。深化财政体制改革，强化预算管理和监督，形成统一规范的财政体制，提高财政资金使用绩效。突出公共财政保障重点，把更多的财政资源用于“三农”、环保、民生和社会事业。

　　全面提高对外开放水平。优化外贸出口结构，扩大自主知识产权、高新技术和机电产品出口，争创国家服务外包示范城市。完善定向招商工作机制，大力引进世界500强项目、地区总部和战略性新兴产业龙头项目，更加注重引进国外先进技术、管理理念和高端人才。加强对企业“走出去”的指导和服务。把握两岸经济合作框架协议实施的机遇，深化青台经贸、文化等领域的交流合作。继续发挥外事工作在对外开放中的重要作用，做好侨务工作。全面加强对口帮扶交流，不断拓展区域合作领域，健全支持内资企业发展的服务网络，积极引进中央企业和知名企业来青发展。

　　（十）加快建设法治政府和服务型政府

　　各位代表！政府的一切权力来自人民，必须对人民负责，受人民监督，让人民满意。做好今年和未来五年经济社会发展工作，必须坚持依法行政、从严治政，加快建立职责法定、程序规范、高效廉洁的法治政府，建立服务市民、服务基层、服务企业的服务型政府。

　　全面推进依法行政。深入落实国务院关于加强法治政府建设的意见。进一步加强行政程序制度建设，强化行政问责，把公众参与、专家咨询、合法性审查、集体讨论决定作为必经程序加以规范，使行政权力在有效监督下运行。认真执行市人大及其常委会的决议，坚持重大事项、依法行政情况报告制度，加强与市政协的民主协商。主动听取民主党派、工商联和无党派人士的意见。自觉接受市人大及其常委会的法律监督和工作监督，主动接受市政协的民主监督，高度重视舆论监督和社会公众监督。强化行政审批、公共资源交易的监督管理，适时清理行政审批事项，落实执法人员资格管理等监督机制。实施“六五”普法规划，做好法治城市创建工作。

　　创新政府服务和政务公开。以建设服务型政府为目标，做好政府机构改革后续工作，完善政府服务体系，增强政府服务功能，扩大公共卫生、大众教育、养老服务等领域政府购买基本公共服务范围。提升电子政务水平，健全政府网上服务体系。深化政府信息公开，高度重视和及时回应社会热点问题，进一步畅通行风在线、政务服务热线、民生在线、纠风网、网络在线问政等沟通渠道，不断完善“向市民报告、听市民意见、请市民评议”活动，健全公众评价机制。

　　切实加强廉政建设。认真落实《党员领导干部廉洁从政若干准则》等各项规定，完善惩治和预防腐败体系。深入推进科技防腐工作，健全行政处罚权、政府投资项目、财政专项资金等网络化监管系统。大力弘扬求真务实、团结协作的工作作风，倡导短、实、新的文风会风。厉行勤俭节约，从严控制一般性支出。充分发挥监察、审计等部门的监督功能，加强对行政权力运行、财政资金使用、经济责任履行的监督，严厉查处违法违纪行为。全体政府工作人员要牢记全心全意为人民服务的宗旨，做出经得起实践、人民、历史检验的业绩。

　　各位代表！“十一五”的辉煌成就令人鼓舞，“十二五”的宏伟目标催人奋进。让我们更加紧密地团结在以胡锦涛同志为总书记的党中央周围，在中共青岛市委的坚强领导下，解放思想，开拓创新，扎实工作，为加快建设富强文明和谐的现代化国际城市，创造全市人民更加幸福美好的未来而努力奋斗！
